# Systematic Analysis of REBASE Identifies Numerous Type I Restriction-Modification Systems with Duplicated, Distinct *hsdS* Specificity Genes That Can Switch System Specificity by Recombination

**DOI:** 10.1128/mSystems.00497-20

**Published:** 2020-07-28

**Authors:** John M. Atack, Chengying Guo, Thomas Litfin, Long Yang, Patrick J. Blackall, Yaoqi Zhou, Michael P. Jennings

**Affiliations:** a Institute for Glycomics, Griffith University, Gold Coast, Queensland, Australia; b College of Plant Protection, Shandong Agricultural University, Taian City, Shandong Province, China; c School of Information and Communication Technology, Griffith University, Gold Coast, Queensland, Australia; d Queensland Alliance for Agriculture and Food Innovation, The University of Queensland, St. Lucia, Queensland, Australia; University of Wisconsin—Madison

**Keywords:** *hsdS*, phase variation, phasevarion, restriction-modification

## Abstract

*N*^6^-Adenine DNA methyltransferases associated with some Type I and Type III restriction-modification (R-M) systems are able to undergo phase variation, randomly switching expression ON or OFF by varying the length of locus-encoded simple sequence repeats (SSRs). This variation of methyltransferase expression results in genome-wide methylation differences and global changes in gene expression. These epigenetic regulatory systems are called phasevarions, phase-variable regulons, and are widespread in bacteria. A distinct switching system has also been described in Type I R-M systems, based on recombination-driven changes in *hsdS* genes, which dictate the DNA target site. In order to determine the prevalence of recombination-driven phasevarions, we generated a program called RecombinationRepeatSearch to interrogate REBASE and identify the presence and number of inverted repeats of *hsdS* downstream of Type I R-M loci. We report that 3.9% of Type I R-M systems have duplicated variable *hsdS* genes containing inverted repeats capable of phase variation. We report the presence of these systems in the major pathogens Enterococcus faecalis and Listeria monocytogenes, which could have important implications for pathogenesis and vaccine development. These data suggest that in addition to SSR-driven phasevarions, many bacteria have independently evolved phase-variable Type I R-M systems via recombination between multiple, variable *hsdS* genes.

**IMPORTANCE** Many bacterial species contain DNA methyltransferases that have random on/off switching of expression. These systems, called phasevarions (phase-variable regulons), control the expression of multiple genes by global methylation changes. In every previously characterized phasevarion, genes involved in pathobiology, antibiotic resistance, and potential vaccine candidates are randomly varied in their expression, commensurate with methyltransferase switching. Our systematic study to determine the extent of phasevarions controlled by invertible Type I R-M systems will provide valuable information for understanding how bacteria regulate genes and is key to the study of physiology, virulence, and vaccine development; therefore, it is critical to identify and characterize phase-variable methyltransferases controlling phasevarions.

## Abstract (precis)

Many bacterial species contain DNA methyltransferases that have random on/off switching of expression. These systems, called phasevarions (phase-variable regulons), control the expression of multiple genes by global methylation changes. In every previously characterized phasevarion, genes involved in pathobiology, antibiotic resistance, and potential vaccine candidates are randomly varied in their expression, commensurate with methyltransferase switching. Our systematic study to determine the extent of phasevarions controlled by invertible Type I R-M systems will provide valuable information for understanding how bacteria regulate genes and is key to the study of physiology, virulence, and vaccine development; therefore, it is critical to identify and characterize phase-variable methyltransferases controlling phasevarions.

## INTRODUCTION

Phase variation is the high-frequency, random, and reversible switching of gene expression (1). Many host-adapted bacterial pathogens encode outer-surface features such as iron acquisition systems (2, 3), pili (4), adhesins (5, 6), and lipooligosaccharide (7, 8) that randomly switch expression ON and OFF in a process known as phase variation, mediated by variation in the length of locus associated simple sequence repeats (SSRs) (1). SSR tracts located in the open reading frame of a gene can result in the gene being in frame and expressed (ON) or, due to a frameshift downstream of the SSR tract, out of frame and not expressed (OFF). SSR tracts also occur in the promoter of a number of genes (9–11), and variation in length of these SSRs can lead to ON-OFF switching of gene expression or result in a gradient of high- to low-level expression dependent on the length of the SSR tract (12). Several bacterial pathogens also contain well-characterized cytoplasmic *N*^6^-adenine DNA methyltransferases, which are part of restriction-modification (R-M) systems, that exhibit phase-variable expression. We recently characterized the distribution of SSR tracts in Type III *mod* genes and Type I *hsdS*, *hsdM*, and *hsdR* genes in the REBASE database of R-M systems, and we demonstrated that 17.4% of all Type III *mod* genes (13) and 10% of all Type I R-M systems contain SSRs that are capable of undergoing phase-variable expression (14). Phase variation of methyltransferase expression leads to genome-wide methylation differences, which can result in differential regulation of multiple genes in systems known as phasevarions (phase-variable regulon). Phasevarions controlled by ON-OFF switching of Type III *mod* genes have been studied extensively in the host-adapted bacterial pathogens Haemophilus influenzae (15, 16), *Neisseria* spp. (17), Helicobacter pylori (18), Moraxella catarrhalis (19, 20), and Kingella kingae (21) and have been recently reviewed (22). Although we have recently demonstrated that almost 10% of Type I R-M systems contain SSRs and can potentially undergo phase variation, phase-variable expression of Type I R-M systems has as yet only been demonstrated in two species: an *hsdM* gene switches ON-OFF via SSRs changes in nontypeable H. influenzae (NTHi) (7, 23) and an *hsdS* gene phase varies due to SSRs alterations in N. gonorrhoeae (24). The *hsdS* gene in N. gonorrhoeae, encoding the NgoAV Type I system, contains a G_[n]_ SSR tract, with variation in the length of this tract resulting in either a full-length or a truncated HsdS protein being produced, rather than an ON-OFF switch seen with the *hsdM* gene in NTHi and Type III *mod* genes. The full-length and truncated HsdS proteins produced from phase variation of the NgoAV system have differing methyltransferase specificities (24).

**FIG 1:**
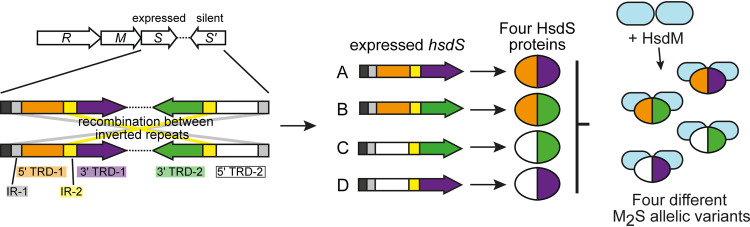
Illustration of how phase-variable switching of inverting Type I systems occurs. Type I R-M loci are made up of three genes encoding a restriction enzyme (*hsdR*; *R*), a methyltransferase (*hsdM*; *M*), and a target sequence specificity protein (*hsdS*; S). Inverting Type I systems contain an extra *hsdS* gene termed *hsdS′* (S′). Each *hsdS* gene is made up of two target recognition domains (TRDs). In inverting systems there are multiple variable TRDs present in the two *hsdS* loci. In the illustrated example, there are two different 5′-TRDs (5′-TRD-1 in orange and 5′-TRD-2 in white) and two different 3′TRDs (3′-TRD-1 in purple and 3′-TRD-2 in green). Inverted repeats are located before 5′-TRD (gray) and between the 5′-TRD and 3′-TRD (yellow). Recombination between these inverted repeats means that four possible *hsdS* coding sequences are present in the expressed *hsdS* locus: allele A = 5′-TRD-1 + 3′-TRD-1; allele B = 5′-TRD-1 + 3′-TRD-2; allele C = 5′-TRD-2 + 3′-TRD-2; allele D = 5′-TRD-2 + 3′-TRD-1. These four different *hsdS* variants mean four different HsdS proteins are produced. Following oligomerization with an HsdM dimer to form an active methyltransferase, the four different HsdS protein subunits result in four different methyltransferase specificities. This would be described as a “four-way” or “four-phase” switch, since four different HsdS proteins are produced from the four different *hsdS* genes possible in the expressed *hsdS* locus.

Type I *hsdS* genes can also undergo phase-variation by recombination between inverted repeats (IRs) encoded in multiple distinct copies of *hsdS* genes encoded in the Type I R-M locus (25; reviewed in reference 26) (Fig. 1). These systems have been named “inverting” Type I loci, since they phase vary via “inversions” between the IRs located in the multiple variable *hsdS* genes. The generation of sequence variation by shuffling between multiple protein variants through IR recombination is perhaps best studied in *pilE* gene encoding pili in N. gonorrhoeae (27, 28) and N. meningitidis (29). In these systems, recombination between a single expressed locus, *pilE*, and multiple adjacent, silent copies of the gene, *pilS*, generate PilE pilin subunit proteins with distinct amino acid sequences. In Type I R-M systems, each HsdS specificity protein is made up of two “half” target recognition domains (TRDs), with each TRD contributing half to the overall specificity of the HsdS protein (Fig. 1A). Therefore, changing a single TRD coding region will change the overall specificity of the encoded HsdS protein. The first example of a phasevarion controlled by an inverting Type I R-M system was described in the major human pathogen Streptococcus pneumoniae strain D39 (25), and subsequent studies have been conducted in strain TIGR4 (30). This system contains multiple variable *hsdS* loci with inverted repeats and a locus-encoded recombinase and switches between six alternate HsdS proteins that encode six different methyltransferase specificities (25) and control six different phasevarions. We recently demonstrated the presence of an inverting Type I R-M system in Streptococcus suis that switches expression between four alternate HsdS subunits (31). The presence of other inverting Type I systems containing multiple variable *hsdS* genes has also been observed *ad hoc* in several bacterial species, including Porphyromonas gingivalis and Tannerella forsythia (26, 32). In this study, we carried out a systematic study of the “gold standard” restriction enzyme database REBASE using a purpose-designed program to systematically identify IRs in *hsdS* genes in order to determine the prevalence of inverting Type I systems in the bacterial domain.

## RESULTS

### A systematic search of REBASE reveals that approximately 6% of all Type I R-M systems contain duplicated *hsdS* loci containing inverted repeats.

**FIG 2:**
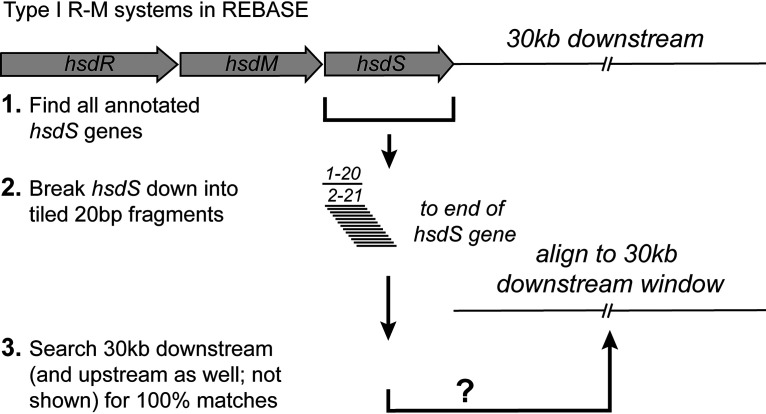
Illustration of our search methodology. All Type I *hsdS* loci were downloaded from REBASE. These loci were then broken down into 20-bp tiled fragments, each staggered by 1 bp (fragment 1 = bp1-20, fragment 2 = bp2-21, etc.). These tiles were then used as a search term to search for 100% identical fragments in the opposite orientation, i.e., inverted, 30 kb upstream of the annotated start codon and 30 kb downstream of the annotated stop codon of the *hsdS* gene under investigation. Although we searched both upstream and downstream of the annotated *hsdS* gene understudy, we have only shown the downstream search in this illustration for simplicity.

In order to identify all Type I *hsdS* genes containing IRs, we searched the restriction enzyme database, REBASE (33), for *hsdS* genes and then searched within 30 kb of the start and end of the annotated *hsdS* for IRs matching a region of the *hsdS* gene being analyzed (see Fig. 2). Using the 22,107 *hsdS* genes annotated in REBASE (see Data Set S1 in the supplemental material [sequences downloaded on 24 October 2018]), we show that 3,683 of these *hsdS* genes contain at least one ≥20-bp sequence with 100% identity to a region that is inverted (i.e., an IR) and within 30 kb of the *hsdS* gene under analysis (Data Set S2). We strictly set our criteria to only select IRs that were 100% identical, and of a minimum size of 20 bp in length. This rationale was based on the SpnD39III system, which we described in 2014 (25). The SpnD39III locus contains three different IR regions that are 15, 85, and 33 bp in length, encoded within multiple variable *hsdS* genes. Therefore, setting our minimum length criteria for an IR at 20 bp means any IRs detected are above the length shown previously to result in homologous recombination between variable *hsdS* genes. Our limit of 30 kb to search up- or downstream was imposed in order to only identify distinct, individual Type I systems containing multiple *hsdS* genes, rather than identifying *hsdS* genes that are part of different Type I R-M systems.

10.1128/mSystems.00497-20.1 DATA SET S1 All Type I *hsdS* genes downloaded from REBASE. Download Data Set S1, XLSX file, 1.2 MB. Copyright © 2020 Atack et al. 2020 Atack et al. This content is distributed under the terms of the Creative Commons Attribution 4.0 International license.

10.1128/mSystems.00497-20.2 DATA SET S2 All IRs found in *hsd*S genes. Download Data Set S2, XLSX file, 0.4 MB. Copyright © 2020 Atack et al. 2020 Atack et al. This content is distributed under the terms of the Creative Commons Attribution 4.0 International license.

We carried out our search for inverted repeats using a bespoke perl script (irepeat.upstream.pl), which we have made available at https://github.com/GuoChengying-7824/type_I. This script was also implemented as a simple, easy-to-use server called “RecombinationRepeatSearch,” which can be found at https://sparks-lab.org/server/recombinationrepeatsearch/. This software allows a user to input any gene or DNA sequence (e.g., an *hsdS* gene) and by providing the relevant upstream and downstream DNA sequence (e.g., the *hsdS* gene plus 30 kb upstream and downstream as a single sequence), the software is able to locate regions containing inverted repeats (see Fig. 2).

Our analysis showed that of the 3,683 *hsdS* genes containing at least one IR, many *hsdS* genes had more than one downstream IR and so were counted twice (for an *hsdS* gene with two downstream inverted repeats), three times (for an *hsdS* gene with three downstream inverted repeats), and so on. Therefore, in order to determine the number of individual *hsdS* genes with at least one downstream IR, we collated together all identical *hsdS* genes. After this collation, we show that 991 individual Type I R-M loci have *hsdS* genes with *at least* one IR located within 30 kb (Data Set S3). Taking into account all of the *hsdS* genes analyzed (22,107), 875 contain at least one IR in a second, duplicated, variable *hsdS* gene within the same Type I locus. This equates to 3.9% (875/22107) of all *hsdS* genes being potentially phase-variable via recombination and therefore able to control phasevarions.

10.1128/mSystems.00497-20.3 DATA SET S3 All representative *hsdS* genes with IRs. Download Data Set S3, XLSX file, 0.05 MB. Copyright © 2020 Atack et al. 2020 Atack et al. This content is distributed under the terms of the Creative Commons Attribution 4.0 International license.

Our analysis shows that some bacterial species contain a relatively low proportion of examples of strains that have IRs within 30 kb of annotated *hsdS* genes. For example, there are 428 Staphylococcus aureus genomes in REBASE, and of these, only 5 contain an *hsdS* gene with an IR located within 30 kb (Data Set S3); of the 232 Pseudomonas aeruginosa genomes examined, only 1 contained an *hsdS* with an IR found within 30 kb. Detailed analysis of these regions revealed that the IR found within 30 kb of the annotated *hsdS* gene in P. aeruginosa strain SPA01 (accession number LQBU01000001) is only 28 bp long, and although it is possible that inversions do occur between these inverted repeats, the IR is not in a locus annotated as an *hsdS*. Manual examination of the 5 IRs found within 30 kb of annotated *hsdS* genes in S. aureus also do not appear in a second annotated *hsdS* locus. Three of these inverted repeats in S. aureus are >200 bp long (in strains 333, M013, and UCI 48); for example, the IR found within 30 kb of the *hsdS* annotated as S.SauM013ORF1818P in S. aureus strain M013 (accession number CP003166; see Data Sets S1 and S2) is 529 bp long. The S.SauM013ORF1818P locus is itself 531 bp long. It is likely that these two regions are able to recombine and flank a region including genes for a hyaluronate lyase and a metalloproteinase. It was recently demonstrated in S. aureus that recombination between two Type I loci ∼1.26 Mb apart are able to mediate genome inversions (34). It is therefore possible that a small proportion of the large (>200 bp) IRs that we identified in our search (Data Set S2) are part of larger inverting DNA segments and not associated with individual Type I loci that undergo rearrangements between expressed and silent *hsdS* genes contained in a single Type I locus, i.e., not part of inverting Type I R-M systems.

Using the SpnD39III system present in S. pneumoniae, which we identified as the first inverting, phase-variable Type I R-M system, and the first example of a phasevarion in a Gram-positive bacterium (25), we observe that all the 52 annotated genomes of S. pneumoniae that we analyzed (out of the 78 strains listed in REBASE) contain the SpnD39III system (Data Set S3). This confirmed the findings in our 2014 study, where we showed every genome in GenBank (*n* = 262) contained a Type I locus where inverted variable *hsdS* genes were present (25). Our systematic search of REBASE also identified the Type I system in S. suis which we have previously shown to shuffle between four different HsdS proteins (31). These findings serve as a “positive control” for our search methodology, in that it is able to identify systems previously shown to contain IRs and to be phase variable by *ad hoc* searches.

Our search confirms the presence of inverting Type I R-M systems with downstream IRs identified previously. For example, we show that 7 of 15 strains of P. gingivalis with an annotated genome in REBASE contain *hsdS* genes with IRs located within 30 kb and that 2 of 7 strains of *T. forsythia* contain annotated *hsdS* genes where IRs are present within 30 kb (32). Our analysis of these regions confirmed the IRs to be present in a second, variable a *hsdS* gene that is part of the same Type I R-M locus, and which we class as an inverting, i.e., a phase-variable Type I locus. Using these systems as an example, and based on previous work with the SpnIII system in S. pneumoniae (25) and the inverting Type I system in S. suis (31), we analyzed the regions immediately upstream of both *hsdS* genes present in each individual P. gingivalis and *T. forsythia* Type I locus containing IRs. This analysis demonstrated that only the *hsdS* gene immediately downstream of the *hsdM* gene is a functional open reading frame, with the second downstream *hsdS* gene encoded on the opposite strand being silent (*hsdS*′), since this second gene does not contain an ATG start codon or a region recognized as a promoter using the bacterial promoter prediction tools CNNpromoter_b (35) and PePPER (36).

### Three major veterinary pathogens contain Type I R-M systems containing duplicated variable *hsdS* loci.

**FIG 3:**
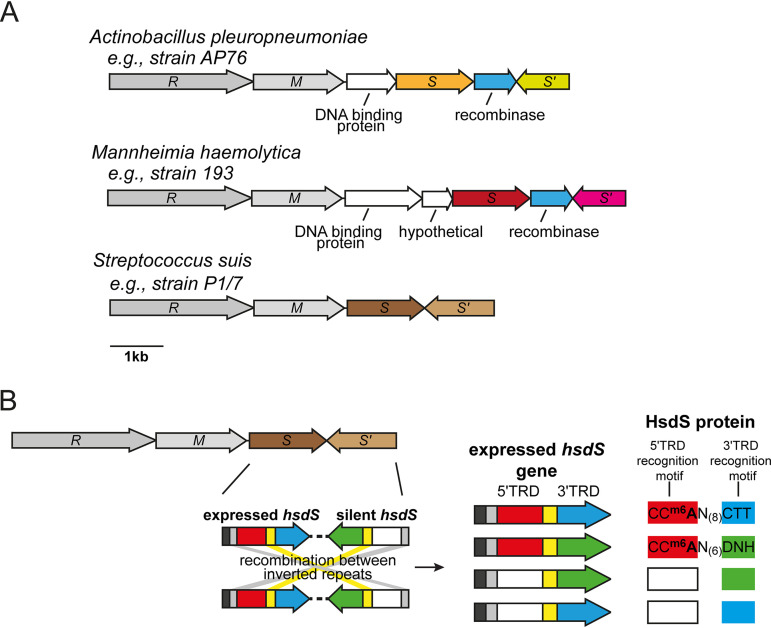
(A) schematic representation of Type I loci with multiple variable *hsdS* genes containing inverted repeats from three important veterinary pathogens. Colored arrows represent variable *hsdS* genes. Each *hsdS* gene is made up of two separate target recognition domains (TRDs; the 5′-TRD and the 3′-TRD), as illustrated in our representative example shown in Fig. 1 and represented in the S. suis example shown in panel B. Blue arrows indicate that a gene with high identity to a recombinase/integrase is present at the locus. (B) Illustration of the mode of switching of the four-way switch occurring in Streptococcus suis. S. suis contains a Type I locus containing duplicated variable *hsdS* loci containing inverted repeats (SSU1271-SSU1274 in S. suis strain P1/7). As illustrated in Fig. 1, each *hsdS* gene is made up of separate 5′ (red and white)- and 3′ (blue and green)-TRDs. Inverted repeats are present before the 5′-TRD (gray) and between the 5′- and 3′-TRDs (yellow). Each TRD recognizes a different 3-bp DNA sequence, giving rise to four separate HsdS proteins that are predicted to methylate four different DNA sequences dependent on the TRDs present. We have solved the specificity of allele A (5′-TRD-1 [red] + 3′-TRD-1 [blue]) and allele B (5′-TRD-1 [red] + 3′-TRD-2 [green]). 5′-TRD-1 (red) recognizes CCA, 3′-TRD-1 (blue) recognizes CTT, 3′-TRD-2 (green) recognizes DNH. D = A, G, or T; N = any nucleotide; H = A, C, or T. XXX = the recognition motif is undetermined.

Many species contained a high prevalence strains with *hsdS* genes with downstream IRs, and with these IRs located within a separate, variable *hsdS* genes that were part of the same Type I locus containing the *hsdS* gene under study. For example, we identified Type I R-M systems with multiple *hsdS* genes in two major veterinary pathogens, in addition to the one identified in S. suis (Fig. 3A; see Data Set S3 in the supplemental material). In the pig pathogen, Actinobacillus pleuropneumoniae, of the 23 genomes available in REBASE, 18 contain at least one Type I R-M system with multiple, variable inverted *hsdS* loci, and with these *hsdS* genes containing the IRs identified by our search. In the cattle pathogen Mannheimia haemolytica, 19 of the 23 strains surveyed contain at least one Type I R-M system with multiple, variable inverted *hsdS* loci with IRs. Detailed examination of each of the inverting Type I R-M systems we identified in A. pleuropneumoniae and *M. haemolytica* showed that these systems also contain a gene encoding a recombinase/integrase and additional genes encoding proteins unknown functions (Fig. 3A). In addition, our survey demonstrated that 24 of 42 S. suis strains analyzed contain an inverting Type I system, confirming our earlier observation that the Type I system in this species is not present in all strains but conserved within a virulent lineage that causes zoonotic infections (31). In all three of these veterinary pathogens, two IRs are present in a second distinct *hsdS* gene (*hsdS*′) immediately downstream of the *hsdS* understudy and part of the same Type I R-M locus (Fig. 1). Examination of the location of each pair of IRs present in these two *hsdS* genes demonstrated that they occurred upstream of the 5′-TRD and between the 5′-TRD and 3′-TRD (Fig. 1 and 3). The presence of multiple IRs that are in a second variable *hsdS* gene (*hsdS*′) immediately downstream of the *hsdS* gene under study is highly suggestive that these *hsdS* genes undergo inversions, i.e., they are phase variable.

We cloned and overexpressed two *hsdS* alleles, alleles A and B, of the Type I inverting system that we found in S. suis (31) in order to solve the methyltransferase specificity of the Type I methyltransferases containing these HsdS proteins. We have used the approach of heterologous expression of methyltransferases in E. coli, coupled to PacBio Single-Molecule, Real-Time (SMRT) sequencing extensively with Type III *mod* genes in order to solve methyltransferase specificity (5, 13), with the same site observed using the native protein using genomic DNA from the actual species and the overexpressed protein in E. coli (31). We only expressed HsdS alleles A and B since we do not observe any strains of S. suis with annotated genomes where either allele C or allele D (Fig. 3B) is present in the *hsdS* expressed locus immediately downstream of the *hsdM* (31). This approach demonstrated that allele A methylates the sequence CC^m6^AN_(8)_CTT (mean interpulse duration [IPD] ratio = 2.33), and allele B methylates the sequence CC^m6^AN_(6)_DNH (D = A, G, or T; H = A, C, or T; N = any nucleotide; mean IPD ratio = 2.53). This is consistent with allele A and allele B sharing the same 5′-TRD (giving the same half recognition sequence of CCA), but a different 3′-TRD (giving different half recognition sequences of CTT and DNH, respectively) (Fig. 3B). Solving the specificity of the two most common alleles found in the expressed *hsdS* locus of this phase-variable system (31) provides valuable information required to fully characterize the gene expression differences that result from the phase variation of this system.

### The major human and veterinary pathogen *Listeria monocytogenes* contains an inverting Type I R-M system.

**FIG 4:**
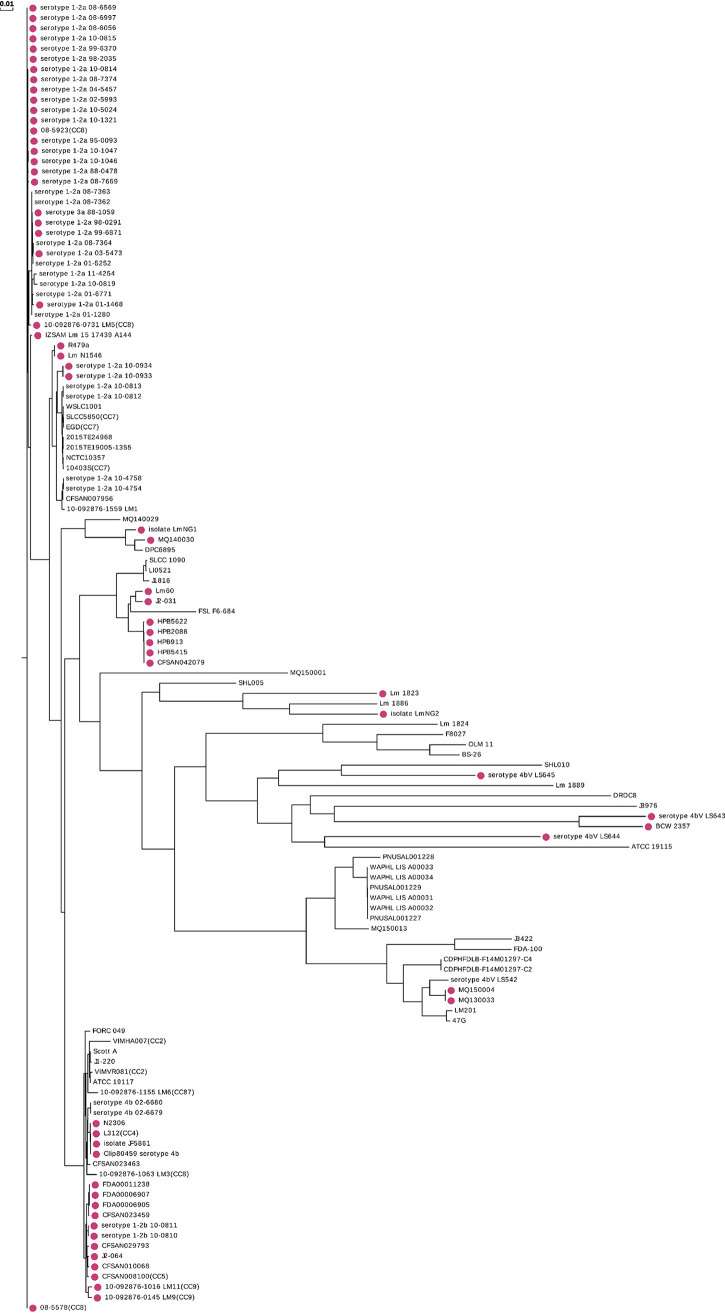
The whole-genome phylogenic tree was constructed by CVTree (version 3.0.0) for 128 strains of Listeria monocytogenes annotated in REBASE. Red circles indicate strains with Type I systems that include duplicated *hsdS* genes containing inverted repeats. The horizontal distance reflects the dissimilarity of each strain.

Our analysis shows that an inverting Type I R-M system is present in approximately half of all strains of Listeria monocytogenes that are deposited in REBASE (60 of 123 strains). This inverting Type I system was previously identified in L. monocytogenes ST8 strains associated with disease in aquaculture and poultry farming (26, 37). Different *hsdS* sequences are present in the expressed *hsdS* locus of multiple strains of L. monocytogenes (37), although no recombination has been demonstrated within an individual strain. Phylogenetic analysis of these strains (Fig. 4) shows that strains containing this system tend to cluster in specific clades. These data suggest that selection and expansion of strains containing this system is occurring, with a possible association between this system and with strains that persist in fish and chickens (37). Analysis of the phenotypes regulated by this system may have an impact on vaccine and pathogenesis studies of this important human and veterinary pathogen.

### The nosocomial, antibiotic-resistant pathogen *Enterococcus faecalis* contains a highly diverse phase-variable Type I R-M locus that is widely distributed.

**FIG 5:**
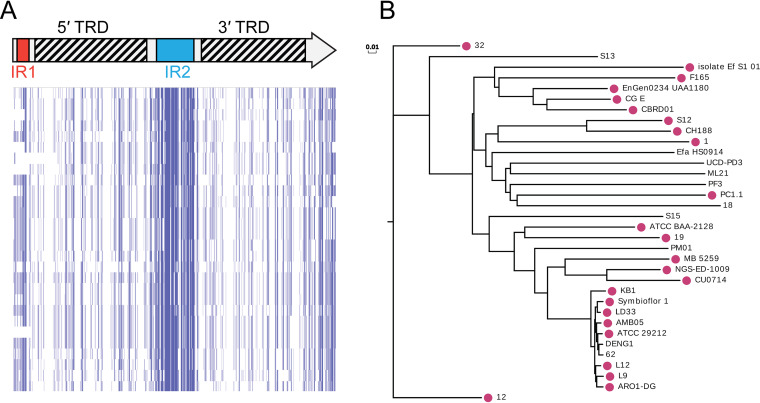
(A) Type I *hsdS* gene showing the location of the 5′- and 3′-TRDs and the inverted repeats. Sequence analysis of representative examples of each *hsdS* gene present in Enterococcus faecalis. Alignments were carried out using ClustalW and visualized in JalView overview feature. Blue color indicates nucleotide identity, with each column representing one nucleotide. (B) The whole-genome phylogenic tree was constructed by CVTree (version 3.0.0) for 34 strains of Enterococcus faecalis annotated in REBASE. Red circles indicate strains with Type I systems that include duplicated *hsdS* genes containing inverted repeats. The horizontal distance reflects the dissimilarity of each strain.

We have been able to identify a Type I R-M system containing multiple variable *hsdS* loci containing IRs present in Enterococcus faecalis, a multidrug-resistant, nosocomial pathogen of major medical importance. This system has been previously noted to occur in a single strain of E. faecalis (26), but no systematic study of the distribution of this system in E. faecalis had been carried out. This system is present in 24 of the 34 strains of E. faecalis present in REBASE. Analysis of the sequences of each of the 24 Type I loci containing duplicated *hsdS* genes (Fig. 5A) shows a high level of variability at each individual *hsdS* locus, with 13 different 5′-TRDs, and 16 different 3′-TRDs present in the *hsdS* genes annotated in REBASE. These data are highly indicative of shuffling of TRDs and shows significant interstrain variability. Our phylogenetic analysis of the strains of E. faecalis containing this system (Fig. 5B) shows that the presence of the Type I R-M system is widely distributed within the overall E. faecalis population and not associated with a particular lineage or groups of strains. This inverting Type I R-M locus also contains an integrase/recombinase, in addition to multiple variable *hsdS* genes containing IRs, adding further weight to the evidence that this system is phase variable.

## DISCUSSION

Our systematic study of REBASE has identified multiple Type I R-M systems that contain inverted repeats that are capable of mediating phase-variable expression and thereby potentially control phasevarions. A previous study demonstrated that integrases/recombinases with high homology to the integrase present in the SpnD39III locus (25) were widespread in the bacterial domain (26). In order to carry out our systematic analysis, we designed software to specifically search for inverted repeats in DNA (code available at https://github.com/GuoChengying-7824/type_I) and applied strict selection criteria so that we only identified inverted DNA repeats that are longer than those that have previously been shown to result in homologous recombination between variable *hsdS* genes (25). We limited the distance away from the *hsdS* locus understudy (30 kb) in order to only identify distinct “inverting” Type I R-M systems. We have made this software available as a user-friendly server version (RecombinationRepeatSearch; https://sparks-lab.org/server/recombinationrepeatsearch/), which allows the user to search any DNA sequence for inverted repeat regions.

By limiting our selection criteria (100% IR identity; minimum IR length of 20 bp; 30-kb window upstream and downstream of each *hsdS*), we have likely missed some Type I loci that are “inverting”; for example, we will miss any IRs that are <20 bp, and we would not detect any *hsdS* containing IRs that are more than 30 kb away. However, we would argue that *hsdS* genes located more than 30 kb away from each other would not comprise a single “inverting” Type I *hsd* locus and that the recombination of these separate *hsdS* genes may not control phasevarions. We also identified a small number of large (>200 bp) IRs present within 30 kb of annotated *hsdS* genes, but a manual examination of these systems revealed that the IRs are not present in a second *hsdS* gene.

Our systematic analysis of REBASE identified Type I loci containing multiple *hsdS* genes where we detect IRs in a range of commensal organisms, such as Bacteroides fragilis and multiple *Ruminococcus* species, in environmental bacterial species such as Leuconostoc mesenteroides and in a number of *Lactobacillus* species that are important to the biotechnology and food production industries (Data Set S3). This reflects our previous studies where we observed simple sequence repeats that mediate phase variation in multiple Type I (14) and Type III methyltransferase genes (13) present in a variety of commensal and environmental organisms.

One obvious reason for generating diversity in methyltransferase specificity is that it will increase resistance to bacteriophage. However, in every case where a methyltransferase has been demonstrated to phase vary, it has also been shown to comprise a phasevarion. Therefore, in addition to improving survival when exposed to bacteriophage, phase-variable methyltransferases are also likely to increase the phenotypic diversity present in a bacterial population, providing bacteria that encode them an extra contingency strategy to deal with changing environmental conditions. It will be interesting to determine how such plasticity of gene expression would be advantageous in a changing environment that cannot be dealt with via conventional “sense and respond” gene regulation strategies (1), particularly as regards phage resistance.

We identified multiple variable *hsdS* loci that contain IRs in the major human pathogens L. monocytogenes and E. faecalis. Our analysis also demonstrated that a variety of veterinary pathogens, contain Type I systems where IRs are present in multiple variable *hsdS* genes. Many of the veterinary pathogens that we show contain inverting Type I loci also contain separate, distinct Type III or Type I R-M systems that are capable of phase varying via changes in locus located simple sequence repeats. These species include Actinobacillus pleuropneumoniae, Mannheimia haemolytica, Streptococcus suis, *Glaesserella* (*Haemophilus*) *parasuis*, and multiple *Mycoplasma* species (13, 14). This means that all of these veterinary pathogens have evolved phase variation of both Type I and Type III methyltransferases, and in the case of Type I systems, by both SSR tract length changes (14) and by recombination between variable *hsdS* genes containing IRs (this study). For example, A. pleuropneumoniae encodes two distinct Type III methyltransferase (*mod*) genes containing simple sequence repeats (13), and a Type I system containing variable *hsdS* loci where IRs are present (this study; Fig. 3A). We predict that this inverting Type I system switches between four separate *hsdS* genes (two separate 5′ TRDs, two separate 3′ TRDs; see the illustration in Fig. 1) and therefore results in four different methyltransferase specificities. Therefore, taking all the possible Type III *mod* ON and OFF combinations (two independently switching Type III *mod* genes [e.g., *mod* genes Y and Z] and therefore four combinations: i, both ON; ii, both OFF; iii, Y ON and Z OFF; and iv, Y OFF and Z ON), together with the four separate HsdS proteins possible (alleles A, B, C, or D), this means that there are a total of 16 different combinations of methyltransferase activity potentially present in a population of A. pleuropneumoniae (four *mod* combinations multiplied by four different *hsdS* alleles; individual bacterial cells will therefore contain one of the *mod* combinations [*n* = 4] and one of the *hsdS* alleles [*n* = 4], so in the population as a whole, *n* = 16 total *mod* + *hsdS* combinations are possible). Therefore, it is critical to determine the genes and proteins that are part of the phasevarions in these species, although this will not be a simple task due the breadth and diversity of the variable methyltransferases present in these organisms.

In summary, we identify that 3.9% of Type I R-M systems contain duplicated variable *hsdS* genes containing inverted repeats, are likely to phase vary, and could consequently control expression of multiple genes due to resulting differential methylation, i.e., control a phasevarion. A broad range of bacterial species encode these systems. Our previous work showed that 2% of Type I *hsdM* and 7.9% of Type I *hsdS* genes contain SSRs (14). Together with our findings in this study, this means that 13.8% of all Type I systems are capable of phase-variable expression. In addition, previous studies have shown that 17.4% of Type III methyltransferases contain SSRs (13) and therefore capable of phase varying. The fact that approximately the same percentage of two independent DNA methyltransferase systems have evolved the ability to phase vary in expression demonstrates that generating variation via switching of methyltransferase expression is a widespread strategy used by bacteria and that this method of increasing diversity has evolved independently multiple times (1). The study of phasevarions is not only key to vaccine development against pathogenic bacteria that contain them but necessary to understand gene expression and regulation in the bacterial domain.

## MATERIALS AND METHODS

### REBASE survey and bioinformatics.

All gene sequences of Type I *hsdS* subunits were downloaded from http://rebase.neb.com/rebase/rebase.seqs.html (24 October 2018). The annotation for each gene was downloaded from http://rebase.neb.com/rebase/rebadvsearch.html. A total of 22,107 genes were obtained with complete annotation information, which includes the start, end, and genomic information of the gene. However, the annotation does not contain the information regarding if the gene is in the positive or the negative strand of the genome. This information is obtained after aligning the gene sequence with the corresponding genomic sequence. All genomic sequences were downloaded from NCBI GenBank, and a total of 15,486 genomes were downloaded. After a gene is located in the corresponding genome, we obtained both 30 kb upstream of the annotated start codon and 30 kb downstream of the annotated stop codon. The 30-kb upstream and downstream regions were compared against 20- to 500-bp fragments of the reverse gene sequence. No reverse search is performed if a gene is in the negative strand. If upstream and downstream regions contain a region mapping to a 500-bp reverse fragment, we further scanned the fragment length between 500 and 1500 bp. This process is implemented by a perl script (irepeat.upstream.pl) located at https://github.com/GuoChengying-7824/type_I. Version 1.0 of the software was used to carry out our search. We also established this software as a server called RecombinationRepeatSearch, and it is located at https://sparks-lab.org/server/recombinationrepeatsearch/. This allows a user to input their gene of interest and, by including the respective upstream or downstream genomic sequence, they are able to determine whether the DNA sequence of their gene of interest encodes inverted DNA repeats in the immediate vicinity.

After this search, all redundant repeating segments were removed by filtering. Only 100% matches for inverted repeats are recorded. All inverted repeat regions found are listed in Data Set S2 in the supplemental material. Phylogenetic trees were constructed using the neighboring method (neighbor joining) using CVTree (version 3.0.0) (38, 39), with the default Hao method, and a K value of 6, as recommended for prokaryotic trees (40).

### Cloning and overexpression of the phase-variable Type I system from *Streptococcus suis*.

The entire *hsdMS* region from S. suis strain P1/7 containing *hsdS* allele B was cloned using primers SsuT1-oE-F (5′-AGTCAG CCATGG GG TCA ATT ACA TCA TTT GTT AAA CGA ATA CAA G) and SsuT1-oE-R (5′-AGTCAG GGATCC TCA GTA ATA AAG TTG GGC AAC TTT TTC) into the NcoI-BamHI site of vector pET15b (Novagen). In order to generate *hsdS* allele A, 3′-TRD allele 1 was synthesized as a gBLOCK (IDT) and cloned into pET15b::allele B that was linearized either side of 3′-TRD allele 2 using the primers TRD-Swap-inv-F (5′-CTG CTG CCA CCG CTG AGC AAT AAC TAG C) and TRD-Swap-inv-R (5′-CTT CCC ATA AGG AGA GTT ATC ATC TCC) to generate vector pET15b::allele A. Inverse PCR using this construct was carried out with KOD polymerase (EMD Millipore) according to the manufacturers’ instructions. After sequencing to confirm the constructs were correct, overexpression of each methyltransferase (HsdM plus either HsdS allele A or HsdS allele B) was carried out using E. coli BL21 cells, which were induced by the addition of IPTG (isopropyl-β-d-thiogalactopyranoside) to a final concentration of 0.5 mM overnight at 37°C with shaking at 200 rpm. Overexpression was confirmed using SDS-PAGE in comparison to an uninduced control.

### SMRT sequencing and methylome analysis.

Genomic DNA from E. coli cells expressing the S. suis HsdM plus either allele A or allele B HsdS were prepared using the Sigma GenElute genomic DNA kit according to the manufacturer’s instructions. Single-Molecule, Real-Time (SMRT) sequencing and methylome analysis was carried out as previously (41, 42). Briefly, DNA was sheared to an average length of approximately 10 to 20 kb using g-TUBEs (Covaris, Woburn, MA), and SMRTbell template sequencing libraries were prepared using sheared DNA. DNA was end repaired and then ligated to hairpin adapters. Incompletely formed SMRTbell templates were degraded with a combination of exonuclease III (New England Biolabs, Ipswich, MA) and exonuclease VII (USB, Cleveland, OH). Primer was annealed, and samples were sequenced on a PacBio RS II (Menlo Park, CA) using standard protocols for long insert libraries. SMRT sequencing and methylome analysis were carried out using SNPSaurus (University of Oregon).
